# Stress and strain propagation on infant skull from impact loads during falls: a finite element analysis

**DOI:** 10.1080/23335432.2020.1719196

**Published:** 2020-01-28

**Authors:** F.J. Burgos-Flórez, Diego Alexander Garzón-Alvarado

**Affiliations:** a Biomimetics Laboratory, Instituto De Biotecnología, Universidad Nacional De Colombia, Bogotá, Colombia; b Mathematical Modelling and Numerical Methods Research Group (GNUM), Universidad Nacional De Colombia, Bogotá, Colombia; c Rational Use of Energy and Preservation of the Environment Group (UREMA), Universidad Del Norte, Barranquilla, Colombia

**Keywords:** Head injury, traumatic brain injury, finite elements method, stress, strain, skull impact, infant falls

## Abstract

**Background and Objective**: To simulate infant skull trauma after low height falls when variable degrees of ossification of the sutures are present. **Methods**: A finite elements model of a four-week-old infant skull was developed for simulating low height impact from 30 cm and 50 cm falls. Two impacts were simulated: An occipito-parietal impact on the lambdoid suture and a lateral impact on the right parietal and six cases were considered: unossified and fully ossified sutures, and sagittal, metopic, right lambdoid and right coronal craniosynostosis. **Results**: 26 simulations were performed. Results showed a marked increase in strain magnitudes in skulls with unossified sutures and fontanels. Higher deformations and lower Von Mises stress in the brain were found in occipital impacts. Fully ossified skulls showed less overall deformation and lower Von Mises stress in the brain. Results suggest that neonate skull impact when falling backward has a higher probability of resulting in permanent damage. **Conclusion**: This work shows an initial approximation to the mechanisms underlying TBI in neonates when exposed to low height falls common in household environments, and could be used as a starting point in the design and development of cranial orthoses and protective devices for preventing or mitigating TBI.

## 1. Introduction

Traumatic brain injury (TBI) is one of the leading causes of mortality and disability in the world (Maas et al. 2008). Worldwide, the mortality rate due to trauma is 19 per 100,000 inhabitants. In Colombia, mortality rates are 125 per 100,000 inhabitants, and approximately 70% of the consultation of emergency services is associated with trauma (Santacruz and Herrera 2014). TBI represents at least half of the trauma-related deaths and produces high costs to the healthcare system due to patients treatment and rehabilitation (Moscote-Salazar et al. 2016). A recent Latin American study reported a 37% mortality rate from a total of 484 patients with severe TBI (Bonow et al. 2017). In the United States, health expenditures reach up to one billion dollars yearly, both directly and indirectly, due to loss of labor productivity (Arango-Lasprilla et al. 2010). In the case of children, head injuries are quite common, and TBI is the leading cause of fatalities and pediatric disability in the United States (Viano et al. 1997; Atabaki 2007). The presence of TBI in children under two years of age causes severe neurological morbidity, alterations in cognitive development, and even death (Keenan et al. 2017).

TBI is produced by impact loads on the skull, known as direct injury, or by the acceleration or deceleration of the head without the direct application of load, known as diffuse injury. However, in most cases, a combination of impact loads and acceleration is present (Hardman and Manoukian 2002). The biomechanical impact on brain structures causes the injury of nervous and vascular tissue through two underlying mechanisms, described as primary and secondary lesions. The primary lesion is defined as the set of nervous and vascular lesions that appear immediately as a consequence of TBI and trigger the transmission of energy to the tissue, producing multidirectional deformations in it. These deformations predominantly affect the neurons and to a lesser extent, the glia and cerebral vascular structures, causing contusions and lacerations in the superficial tissue and axon and vascular stretching in the deeper tissue. The secondary lesion refers to the appearance of new lesions in the nervous tissue of a percentage of the patients hospitalized by TBI, which, instead of improving, present deterioration of their condition. These lesions are triggered by a limited number of biochemical chain reactions that progressively affect brain tissue. Thus, the primary lesion tends to get worse, generating final damage more severe than the one directly caused by the primitive impact (Hardman and Manoukian 2002).

As mentioned by (Kraus et al. 1990), the magnitude of the problem has only recently been appreciated with the increase of experimental studies aimed at determining the relationship between biomechanical skull impacts and TBI (Prange et al. 2004; Loyd 2011). On the other hand, with the advent of advances in computational technology, computational numerical studies have been developed to determine relationships between impact kinetics and the distribution of stresses and deformations, which can be correlated with the risk of TBI.

Through the use of the finite element method (FEM), several studies have focused on modeling and computationally simulating the effect of various types of skull impacts on the relative motion and stress and strain distributions of adult brains (Zhang et al. 2001; Yang et al. 2014; Torkestani et al. 2015; Hasnun et al. 2015). However, these studies have not taken into account the intrinsic differences of infant skulls when it comes to experiencing a fall and impact on the encephalic structures. Among these characteristics, the continuous geometric change caused by brain expansion during the first two years of life generates an initial bulge in the parietal and frontal bones, which is not present in the adult skull. In addition, the presence of soft tissues such as sutures and fontanels, primary sites of mechanical deformation and bone formation processes during the first two years of postnatal life, and a single layer of cortical bone less than 2 mm thick in each flat bone of the calvaria, make the child’s skull one of less mechanical resistance when compared to that of an adult. Therefore, a specific biomechanical analysis for the infant’s skull that considers its particularities is necessary to generate a greater understanding of TBE during childhood.

For ethical reasons, few experimental studies have been performed. Among them, Prange et al. (Prange et al. 2004) executed a drop test on heads of newborn specimens from 15 and 30 cm high and considered five impact locations: frontal, occipital, vertex, right, and left parietal. Loyd (Loyd 2011) performed a fall test from 15 and 30 cm on cadavers of newborns of 1, 5, 9, 11, 22 months of age. For both experiments, impact sites and acceleration curves were reported over time. Both studies have been used as a source of validation for computational studies related to trauma from falls in children and neonates.

Several computational studies have developed three-dimensional models of children’s heads to study the impact of falls and their effects on brain structures. (Lapeer and Prager 2001) developed a finite element model of an infant’s head to simulate the deformation of the skull during birth. (Klinich and Hulbert 2002) developed a 3d model of the head of a 6-month-old child, which was subjected to impacts due to the deployment of an airbag during vehicle collisions. Roth et al. have developed various models of children and adults. Among them, a model of the head of a six-month-old child in which they simulated TBI due to shaking and impacts, another of a 3-year-old and one of a 17-year-old, which they used to analyze intracranial injuries and pediatric fractures during reconstruction of accidents with TBI, being the last of these validated experimentally with real TBI data (Roth et al. 2007a, 2007b, 2009; Roth and Raul 2008, 2010). (Coats et al. 2007) developed a 6-week-old infant head model in which they simulated occipital impacts on concrete from 30 cm high. They found that variations in the brain’s comprehensibility greatly affected the stress distribution in the skull, while variations in sutural width and thickness did not have a considerable effect on stresses.

Li et al. (2011) developed a parametric model of the head of an infant between 0 and 3 months of age, in which material properties of the different tissues were varied to find those parameters that had significant effects on the response of the brain. The same research group (Li et al. 2013) subsequently developed a model of the head of a 6 month-old child in which free falls and a compression test were simulated in order to evaluate the effect of different fall heights and surface rigidity on head dynamic response. Results from this work were validated with data from the Loyd experiment (Loyd 2011).

Recently, (Li et al. 2017) developed a finite element model of the head of a newborn and infants of 5 and 9 months of age. A hyperelastic Odgen model was implemented for sutures, non-linear models for scalp and dura mater and orthotropic elasticity constants were included for flat bones. Among their results, better performance in the acceleration-time per impact and force-deflection per compression curves than those previously published in linear elastic models were obtained.

Despite the presence of the exposed computational models, there is still an absence of parametric models of the head that analyze the effect of skull impact from falls when variations in the degrees of ossification of sutures exist. That is fully ossified skulls and those with soft tissue in sutures and fontanels. This case is quite common in patients presenting craniosynostosis, a condition characterized by premature fusion of one or more sutures during prenatal development and first years of life. Similarly, the previous computational studies did not show data concerning stress and strain distributions along with brain tissue when subjected to parietal and occipital impacts and variable degrees of suture ossification.

**Figure 1.**
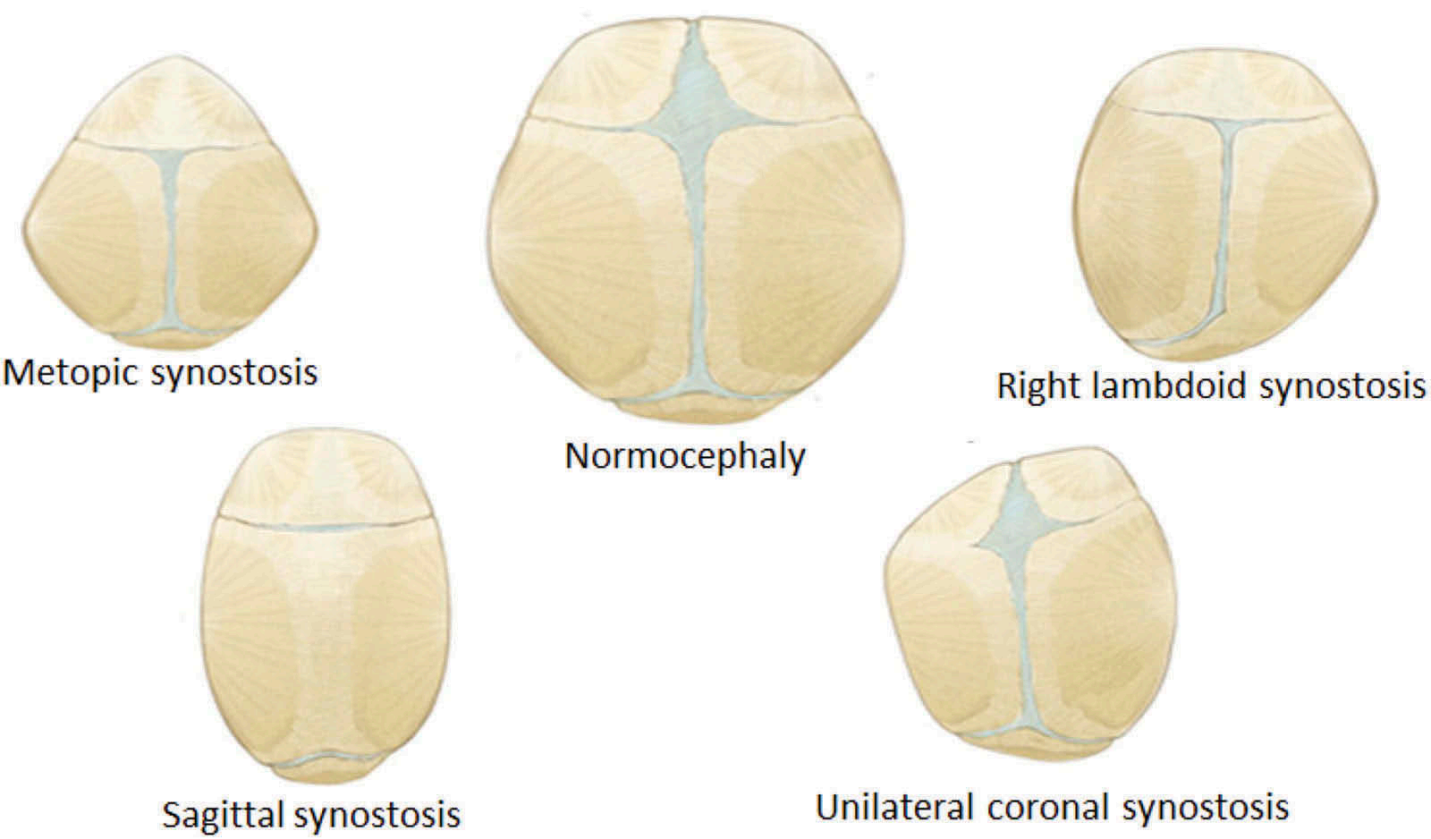
Types of suture ossification considered in this study. Blue regions correspond to non-ossified connective tissue corresponding to sutures and fontanels. Other colors represent bone. The case of fully ossified sutures and fontanels is not represented but was also considered. Skull morphology was maintained constant in this study

The purpose of this work was to simulate infant skull impact produced by different fall heights. For this, a three-dimensional model of the skull of a four week-old infant was developed. Two impact cases were considered in the dynamical analysis: Occipital impact and right parietal impact. These are the most common falls in infants and are presented from heights of 30 and 55 cm (Ainsworth et al. 2014). Stress and strain distribution in soft tissues such as sutures, fontanels, and the brain were studied using the results of the dynamic analysis. Different degrees of ossification were defined for sutures and fontanels in the computational experiment: a) standard non-ossified sutures, b) prematurely fused sutures representing right lambdoid craniosynostosis (premature fusion of right lambdoid suture), sagittal craniosynostosis (premature fusion of the sagittal suture), right coronal craniosynostosis (premature fusion of the right coronal suture), metopic craniosynostosis (premature fusion of the metopic suture) and c) entirely ossified sutures and fontanels (see Figure 1).

This work shows an initial approximation to the mechanisms underlying TBI in neonates, with or without the presence of craniosynostosis, when exposed to low height falls common in household environments. The results obtained from it could be used as a starting point in the design of strategies aimed at mitigating or preventing TBI through the development of cranial orthoses and protective devices.

## 2. Materials and methods

### 2.1. Finite elements model of the infant skull

#### 2.1.1. Head geometry

**Figure 2.**
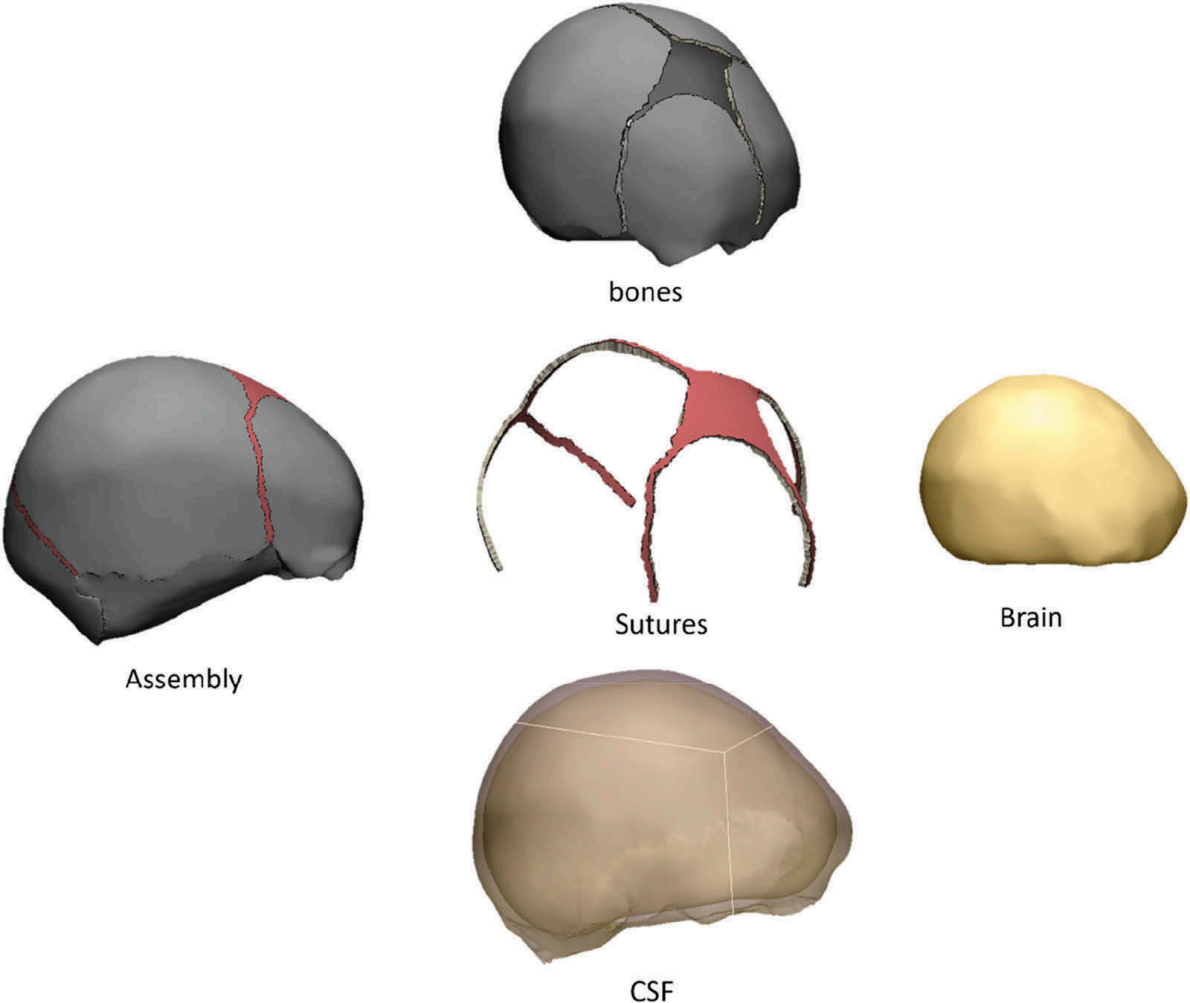
Surface meshes of the neonatal skull model in 3-matic software

The geometry of the head was obtained from an stl file acquired from the company OSTEO3D (Bangalore, India), which came from a three-dimensional reconstruction of the skull using CT images (Osteo3d n.d.). This file contained the three-dimensional geometry of flat bones and bones of the cranial base that form the skull of a newborn baby, in which flat bones were, on average, 1.5 mm thick. Utilizing the software Materialize 3-Matic, it was possible to perform an initial modification of the mesh surfaces using triangular elements. From this initial model that only had flat bones, it was possible to perform 3d modeling of sutures, brain, and fontanels, where each of the meshes corresponded to a hollow volume enclosed by a mesh of unstructured triangles (see Figure 2). The scalp was not modeled to simplify the model and avoid excessive complexity in computational implementation. Also, the morphological interaction of the pia mater, the subarachnoid space, arachnoids, subdural space, and dura mater was not taken into account. Hence, a volume was assumed between the inner surface of the bones, sutures and fontanels and the outer surface of the brain, corresponding to tissue with properties similar to the cerebrospinal fluid (CSF), with a thickness equal to 3 mm. The brain was modeled as a single tissue without making structural differentiations between gray matter, white matter, cerebellum, and brain stem.

#### 2.1.2. Mesh generation

**Figure 3.**
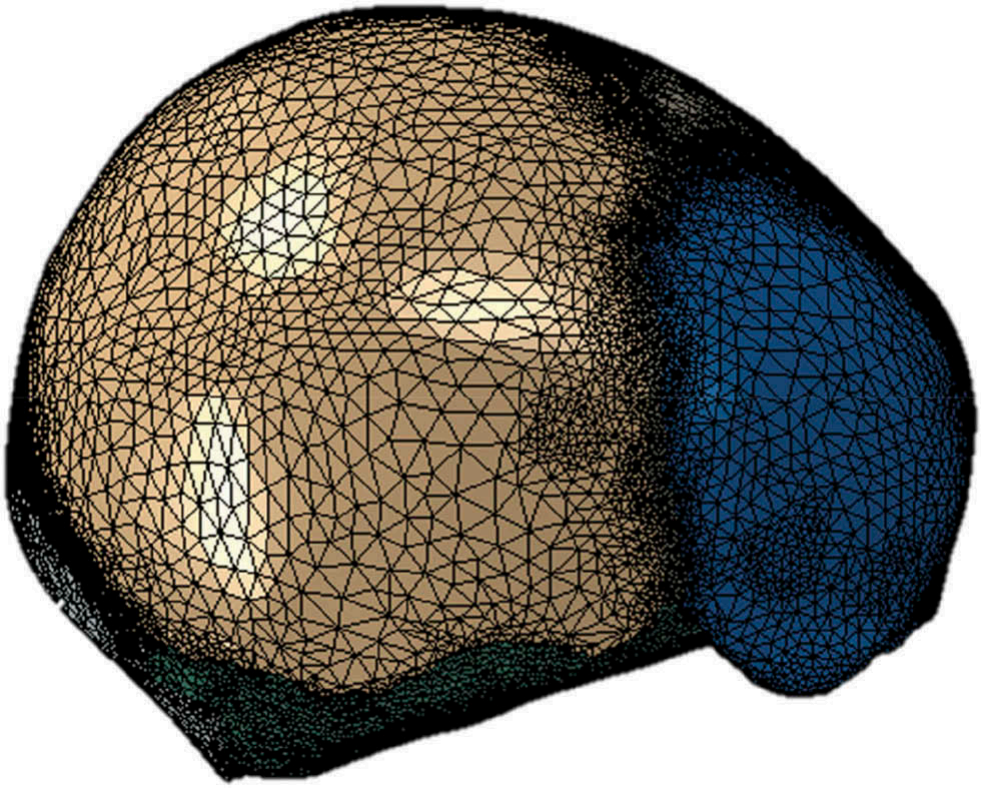
Assembly of the skull model in Abaqus CAE

The software Materialize 3-matic was used for manually creating triangular meshes of the surfaces of each of the specified tissues. From it, closed volumes of each tissue were obtained, thus giving a total of 8 surface meshes corresponding to a left parietal bone, a right parietal bone, frontal bone, occipital bone, sutures with fontanels (a volume for this), cranial base, CSF and brain, as seen in Figure 2. Each surface mesh was exported in .inp format to the ICEM CFD 17.0 meshing software. Using the algorithm of Delaunay, tetrahedral meshes were automatically generated for each volume, maintaining the triangular surface meshes unchanged. Those meshes were proved systematically with different sizes in order to study their convergence. Each of the meshes generated in ICEM was exported in .inp format and imported into the software Abaqus 6.10, where the assembly of the complete head was made, as shown in Figure 3. In total, 100864 nodes and 540248 tetrahedrons were obtained, with an average aspect ratio of 1.78.

#### 2.1.3. Mesh independence analysis

Different meshes with decreasing element size were tested to ensure mesh independence. Among them, a mesh of 690513 elements with an average element axis length of 0.762 mm and a mesh of 540248 elements with an average element axis length of 1.27 mm resulted in brain and skull average Von Mises stresses with less than 5% difference. However, the finest mesh showed a value of stable time increment equal to 1E-009 seconds, significantly increasing computational costs. Conversely, the 540248 elements mesh presented a stable time increment equal to 1E-007 seconds, with a simulation time of 3 hours. Hence, the latter was chosen and used satisfactorily for the simulations, showing stability, low generation of distortions in the elements, fast computational time, and maintained, with very slight variations, the morphological characteristics of the skull geometry.

#### 2.1.4. Model anthropometry

Literature reports were used to develop the geometry of the dynamic and finite element model, where dimensions of the head, skull, and brain mass of a child were determined by direct measurement (Dekaban 1977). The proposed model had an average length of 109 mm, an average width of 113 mm and a volume of 523.5 cubic centimeters, which corresponded to the head of an average newborn of 50 percentile of fewer than four weeks old, according to the table of cranial dimensions reported by Dekaban (Dekaban 1977). The mass obtained for the skull was equal to 629 grams, which is 3.2% lower than the experimental masses of 650 grams used in the study by Prange on the anthropometry of an infant’s head (Prange et al. 2004).

#### 2.1.5. Material properties

Few studies have reported mechanical properties for bony tissues. Hence, there is a lack of reports corresponding to their viscoelastic properties, although the viscoelastic constitutive model could represent more accurately the deformation in the skull in children due to its soft tissue characteristics. Most finite element models have employed isotropic, linear elastic material models to be used for the scalp, skull, sutures, dura, and CSF, and a viscoelastic model for the brain(Brooks et al. 2018). To the best of author’s knowledge, the only experimental study was the one made by Coats and Margulies (Coats and Margulies 2006). In this, linear-elastic properties were defined from tensile tests for bones and sutures.

For these reasons, material properties were simplified to linear elasticity, leaving aside the viscoelastic component. The elastic modulus for the flat bones was defined as the average between the elastic modulus perpendicular to the direction of the fiber and the elastic modulus parallel to the direction of the fiber. These values were defined following the results of previous experimental studies (MCPherson and Kriewall 1980; Coats and Margulies 2006) and taking into account the work by (Brooks et al. 2018).

Brain modeling has significantly varied depending on the age of the human being to be used. For adult brains, hyperelastic materials models such as neo-Hookean (Mihai et al. 2015), Fung (Mihai et al. 2015), Gent (Mihai et al. 2015), Mooney Rivin (Mihai et al. 2015), and Ogden (Kleiven 2007; Patton et al. 2013) have been used. For the brains of children, models of viscoelastic character given by a prony series have been reported (Zhang et al. 2001; Roberts et al. 2012; Yang et al. 2014; Hasnun et al. 2015). In this work, the same pattern was followed, and a viscoelastic model was used to define the material properties of the brain.

Table 1 summarizes the parameters used in the linear elastic model. The majority of these were found in the literature. As observed, isotropic linear-elastic properties were chosen for flat bones, skull base bones, sutures, and fontanels. For the brain and CSF, viscoelastic properties were defined using a time prony series that specified a shear modulus, a compressibility module, and a relaxation time. Furthermore, Young’s module was defined for t = 0 and a Poisson’s ratio close to 0.5 for brain and CSF (brain: 0.49 and CSF: 0.499), considering that these tissues are thought to be almost incompressible. The defined parameter values were chosen considering the inherent individual mechanical variations that occurred in these tissues throughout human development. Nevertheless, since this work is aimed at supporting product development for impact mitigation, the estimation of the initial parameters was deemed adequate for this purpose.

**Table 1.** Material properties and element count for each modeled tissue

Component	Element count	Density ($\frac{Kg}{m^{3}}$)	Elastic behavior (Young’s Module (MPa) and Poisson’s ratio)	Viscoelastic behavior through a Prony series	Reference
Occipital bone	23,515	2150	E=200υ=0.22	– – – – – – –	(Coats and Margulies 2006,MCPherson and Kriewall 1980)
Left parietal bone	39,925	2150	E=200υ=0.22	– – – – – – –	(Coats and Margulies 2006,MCPherson and Kriewall 1980)
Right parietal bone	33,227	2150	E=200υ=0.22	– – – – – – –	(Coats and Margulies 2006,MCPherson and Kriewall 1980)
Frontal bone	65,838	2150	E=200υ=0.22	– – – – – – –	(Coats and Margulies 2006,MCPherson and Kriewall 1980)
Sutures	48,065	1130	E=8.1υ=0.48	– – – – – – –	(Coats et al. 2007,Galford and McElhaney 1970)
Cranial base bones	62,303	2150	E=200υ=0.22	– – – – – – –	(Kriewall 1982,McElhaney et al. 1970)
CSF	128,165	1040	E=0.012υ=0.499	$G_{1}=0.9$$K_{1}=0$$\tau_{1}=50$	(Zhang et al. 2001)
Brain	139,210	1040	E=0.018υ=0.49	$G_{1}=0.61$$K_{1}=0$$\tau_{1}=20$	(Zhang et al. 2001)

#### 2.1.6. Simulations

**Figure 4.**
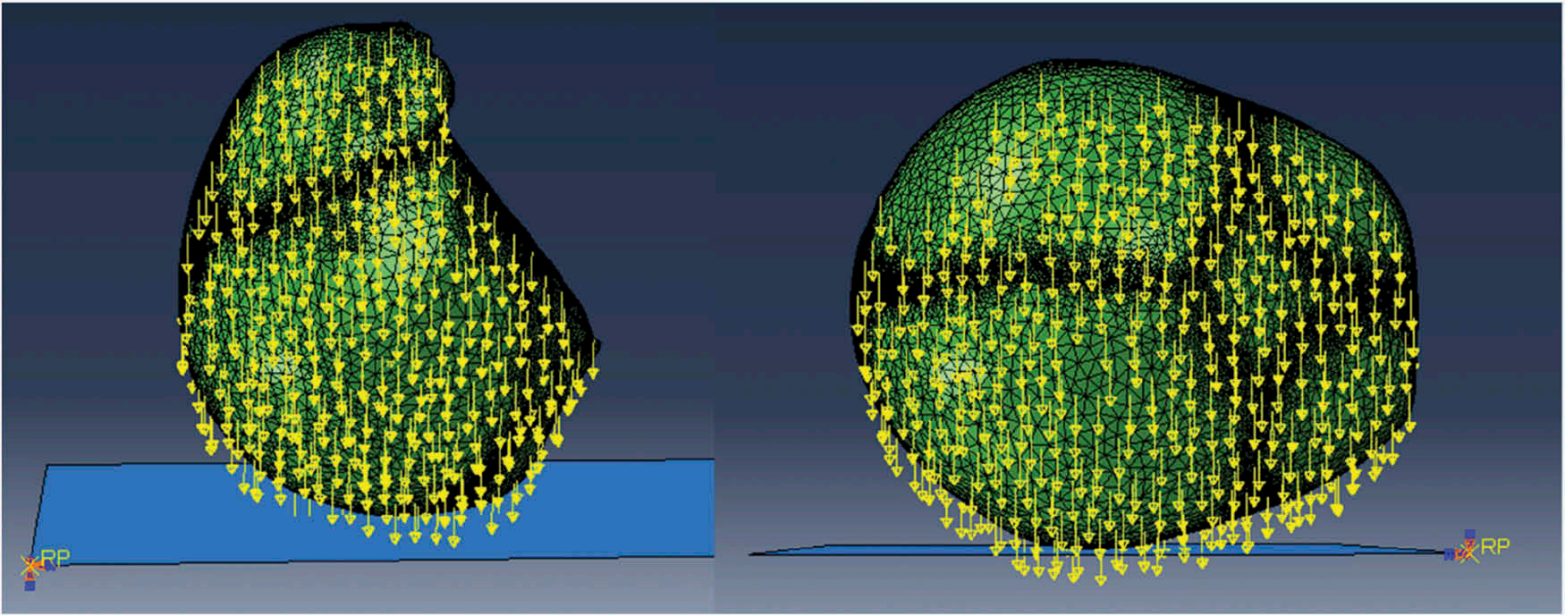
Occipito-parietal and right parietal impacts

A finite element model of the skull of a four week-old infant was developed and employed for simulating free fall from low heights. Two types of impacts were simulated, each considering different fall heights and different degrees of ossification in the suture and fontanels. The first one was an occipito-parietal impact on the lambdoid suture and the second one, a lateral impact on the right parietal bone. Impacts were simulated after a free fall from 30 cm and 50 cm in height. Six different cases of the degree of ossification of sutures and fontanels were established as follows. A first case considered unossified sutures and fontanels, using the material properties defined for sutures in Table 1. A second case considered fully ossified sutures and fontanels. In this case, the mechanical properties of these tissues were assumed to be the same as those of flat bones. Thus, Young’s modulus was equal to 200 MPa, density equal to 2150 $\frac{Kg}{m^{3}}$, and Poisson’s modulus equal to 0.22. Cases three to six considered the premature fusion of the sutures. Four different types of craniosynostosis were considered: sagittal, metopic, right lambdoid, and right coronal craniosynostosis. Therefore, the suture level of ossification was modeled by changing the material properties of the sutures according to each type of craniosynostosis. Skull morphology remained constant since we wanted to compare how the suture’s level of ossification influenced stress and strain propagation in the different tissues forming the infant’s head. Figure 1 provides an overall view of the effects of premature suture fusion on the overall shape of the infant skull.” Two types of impact, two fall heights and six cases of ossification of suture and fontanels, resulted in a total of 24 simulations performed, each one with a simulation time of 8 ms with time steps of 1E-007 seconds. Each simulation took into account non-linear effects in the deformation. Two types of contact between the rigid surface and the skull were defined: A normal contact and a tangential contact in which a coefficient of friction equal to 0.2 was established. The gravity was equal to 9.8 $\frac{m}{s^{2}}$ and, from the equation $V_{0}=\sqrt{2gh}$, where g is the gravity and h the height of the fall, initial skull velocity at impact time was set at 2.42487 $\frac{m}{s}$ and 3.130$\frac{m}{s}$, corresponding to free falls from 30 and 50 cm in height, respectively. These data were previously found in the dynamic model. The impact occurred against a rigid surface with properties similar to those of ceramic material (E = 150 GPa, υ = 0.17), as shown in Figure 4. As in the dynamic model, boundary conditions were defined as no strain, and no external forces were applied to the model before impact.

## 3. Results

**Figure 5.**
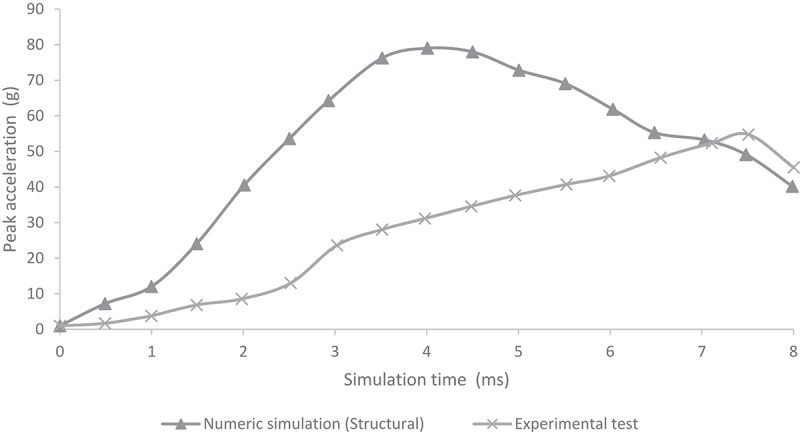
Comparison of numerical peak acceleration results in occipital impact from 30 cm fall with experimental data given by (Prange et al. 2004)

**Figure 6.**
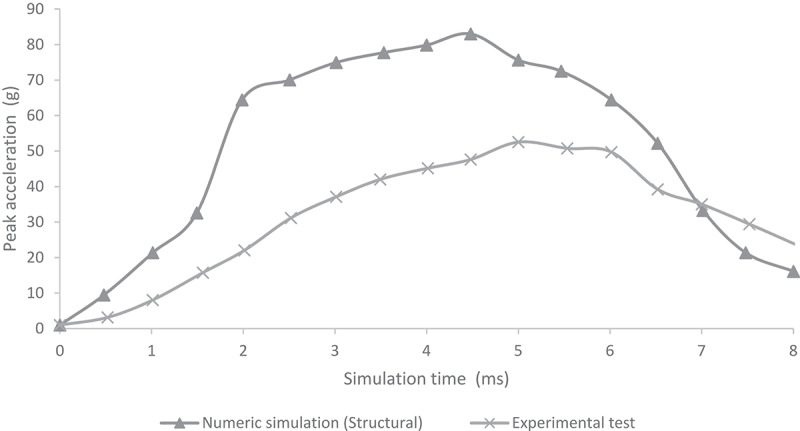
Comparison of numerical peak acceleration results in parietal impact from 30 cm fall with experimental data given by (Prange et al. 2004)

Figures 5–6 show the comparison of peak acceleration values in both occipital and parietal impacts simulation with experimental results obtained from (Prange et al. 2004). Peak accelerations are higher in the numerical simulations of this work in both occipital and parietal impacts when compared to the results obtained by (Prange et al. 2004). The former could be explained by the lack of scalp tissue, skull, and neck muscles, which could provide a damping effect by absorbing deformation energy and reducing acceleration at impact.

### 3.1. Finite elements model

#### 3.1.1. Impact force

**Figure 7.**
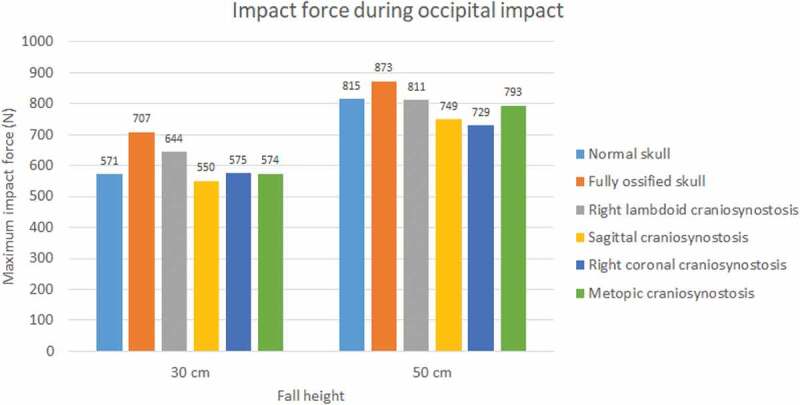
The maximum impact force in occipital impact from 30 and 50 cm falls with different degrees of ossification in the sutures

**Figure 8.**
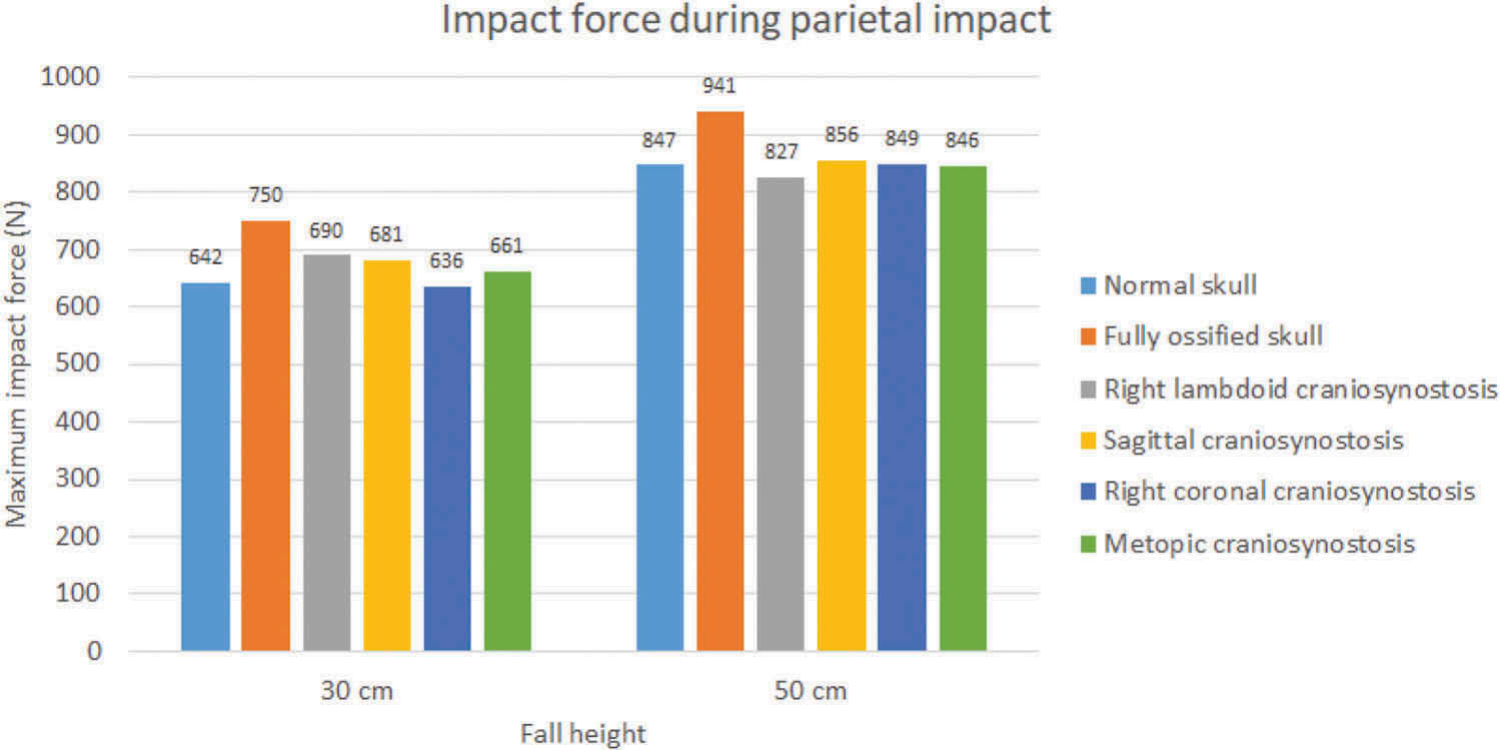
The maximum impact force in parietal impact from 30 and 50 cm falls with different degrees of ossification in the sutures

The impact force was higher in parietal impacts than in occipital ones (see Figures 7–8). Fall height had a positive impact on the impact force in all cases and mainly on the normal skull, in which it increased by almost 43% for the occipital impact. A higher level of ossification generated stronger impacts, with the maximum impact being that of the fully ossified skull (941 N) with parietal impact due to a 50 cm fall. In occipital impact, the lowest values were for cases of sagittal craniosynostosis in 30 cm fall and right coronal craniosynostosis in 50 cm fall. On the other hand, in parietal impact, the lowest impact on 30 cm fall was in right coronal craniosynostosis and, in 50 cm fall, in right lambdoid craniosynostosis.

#### 3.1.2. Flat bones response

**Figure 9.**
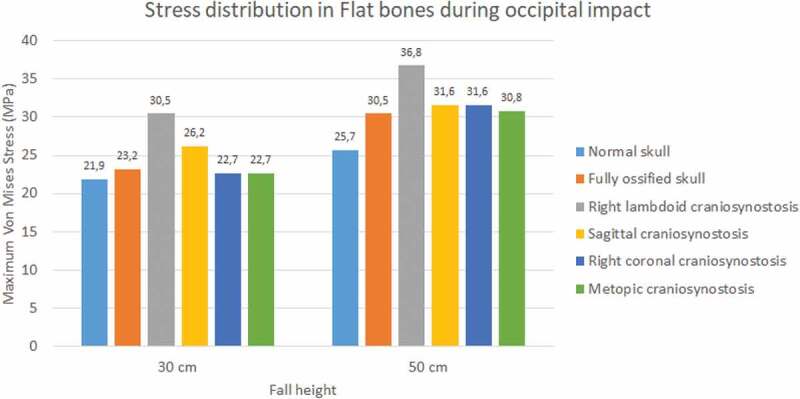
Maximum Von Mises stress in flat bones during occipital impact from 30 and 50 cm falls with different degrees of ossification in the sutures

**Figure 10.**
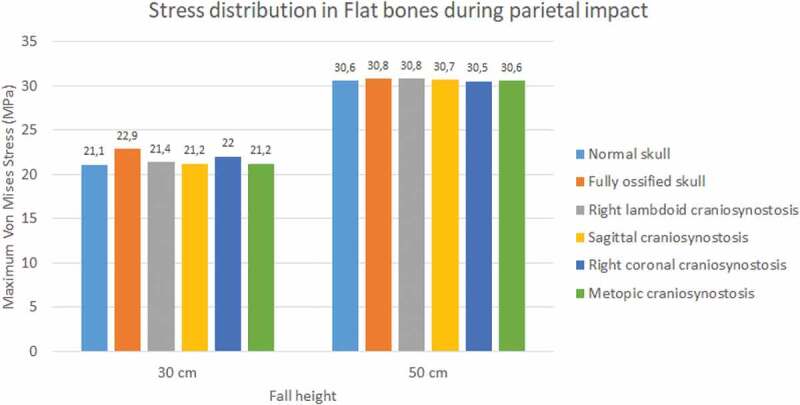
Maximum Von Mises stress in flat bones during parietal impact from 30 and 50 cm falls with different degrees of ossification in the sutures

The occipital impact caused higher values of Von Mises stress and strain than parietal impact (see Figures 9–10). The case with right lambdoid craniosynostosis showed the highest Von Mises stress in occipital impact (30.5 MPa at 30 cm and 36.8 MPa at 50 cm). Occipital impact showed a more variable response than parietal impact, which, in a 50 cm fall, did not generate considerable variations between the different study cases.

**Figure 11.**
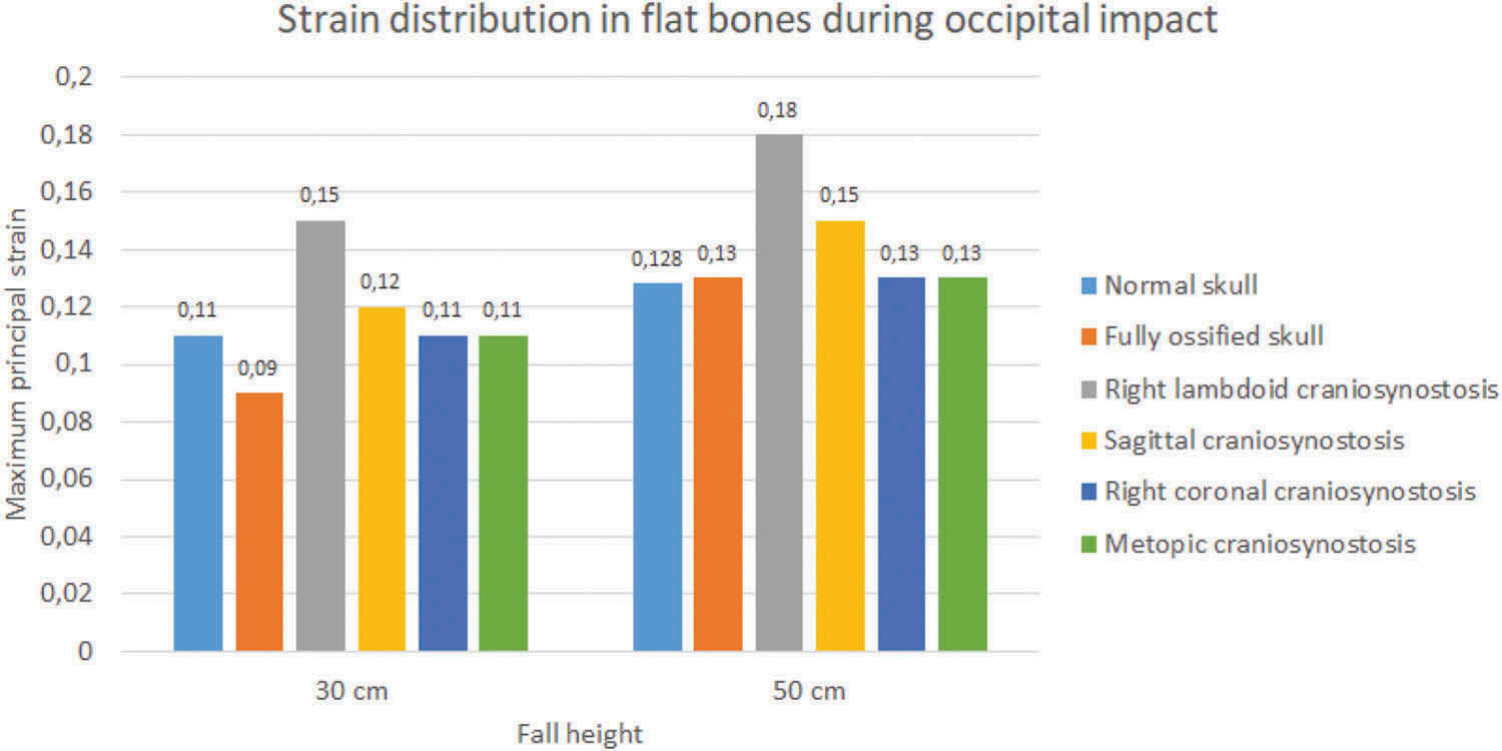
The maximum principal strain in flat bones during occipital impact from 30 and 50 cm falls with different degrees of ossification in the sutures

**Figure 12.**
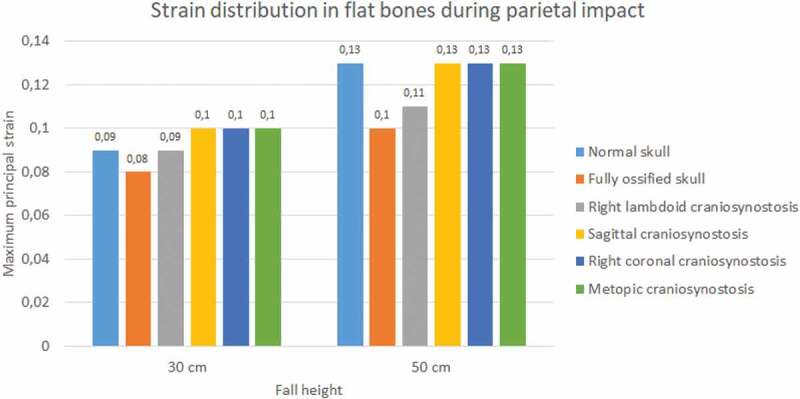
The maximum principal strain in flat bones during parietal impact from 30 and 50 cm falls with different degrees of ossification in the sutures

In the occipital impact, the highest strain was obtained in the case of right lambdoid craniosynostosis (0.15 at 30 cm and 0.18 at 50 cm fall), followed by sagittal craniosynostosis (0.12 at 30 cm and 0.15 at 50 cm fall) (see Figures 11–12). The ossified skull showed the lowest value of deformation in 30 cm fall (0.09) and the normal skull in 50 cm fall (0.128). For the parietal impact, the cases of sagittal, right coronal, and metopic craniosynostosis obtained the highest degree of strain (0.1) in 30 cm fall and in 50 cm fall along with the normal skull.

#### 3.1.3. Sutures response

**Figure 13.**
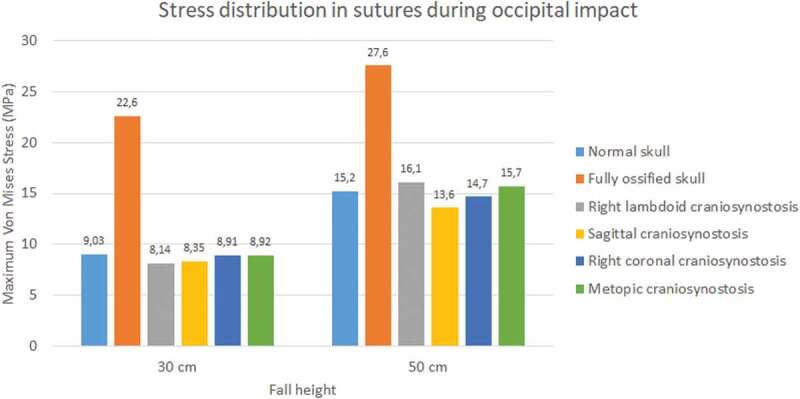
Maximum Von Mises stress in sutures during occipital impact from 30 and 50 cm falls with different degrees of ossification in the sutures

**Figure 14.**
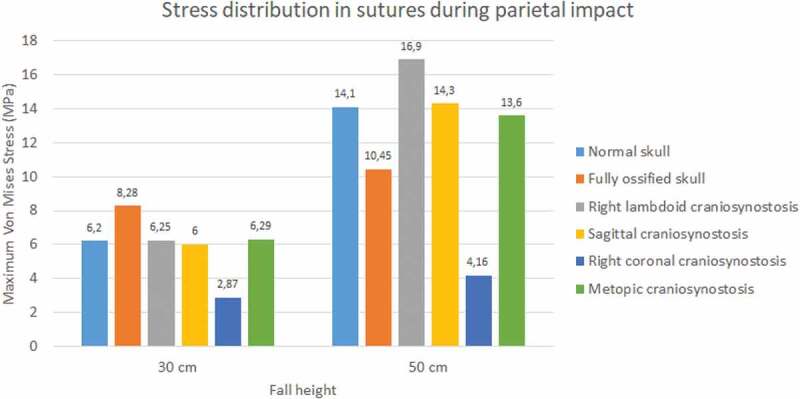
Maximum Von Mises stress in sutures during parietal impact from 30 and 50 cm falls with different degrees of ossification in the sutures

During the occipital impact, the ossified skull showed the highest values of Von Mises stress (see Figures 13–14). This case was expected since the suture was replaced by bone tissue in those cases. The cases of normal skull and skull with metopic craniosynostosis obtained the highest Von Mises stress. In parietal impact, high variations in the results were found. The ossified skull obtained the highest Von Mises stress value in the 30 cm fall (8.28 MPa), but the case with right lambdoid craniosynostosis obtained the highest in 50 cm fall (16.9 MPa). In both fall heights, the case with right coronal craniosynostosis obtained the lowest Von Mises Stress (2.87 at 30 cm fall and 4.16 at 50 cm fall).

**Figure 15.**
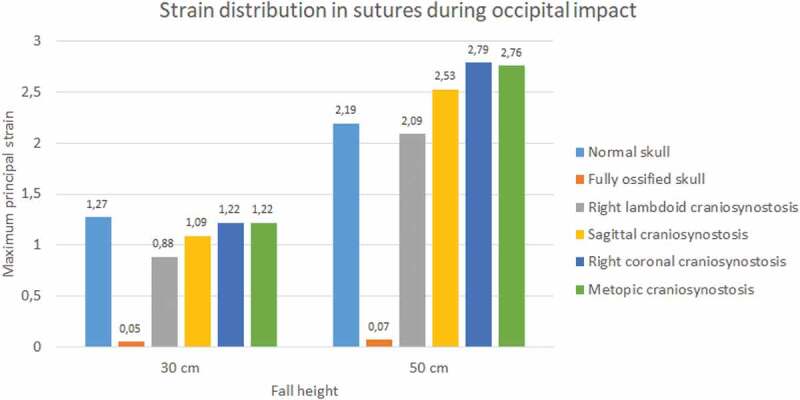
The maximum principal strain in sutures during occipital impact from 30 and 50 cm falls with different degrees of ossification in the sutures

**Figure 16.**
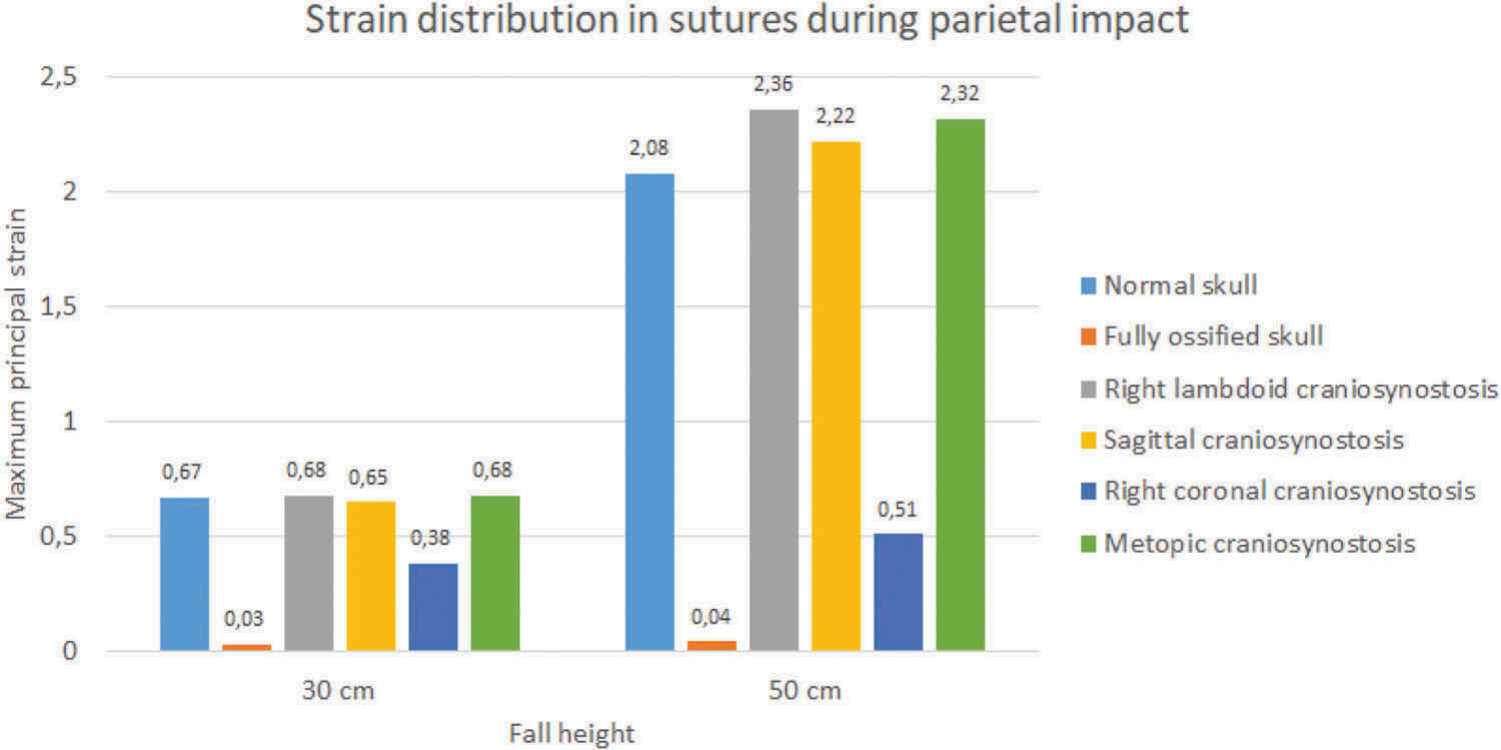
The maximum principal strain in sutures during parietal impact from 30 and 50 cm falls with different degrees of ossification in the sutures

Sutures strain varied significantly. In both impacts and fall heights, the ossified skull showed the lowest values of strain when the sutures were completely ossified (see Figures 15–16). Occipital impact showed the highest strain value for the normal skull (1.27) at 30 cm fall and the skull with right coronal craniosynostosis (2.79) at 50 cm fall. For the parietal impact, the skull with right lambdoid craniosynostosis presented the highest strain value (0.68 at 30 cm fall and 2.36 at 50 cm fall).

#### 3.1.4. Brain response

**Figure 17.**
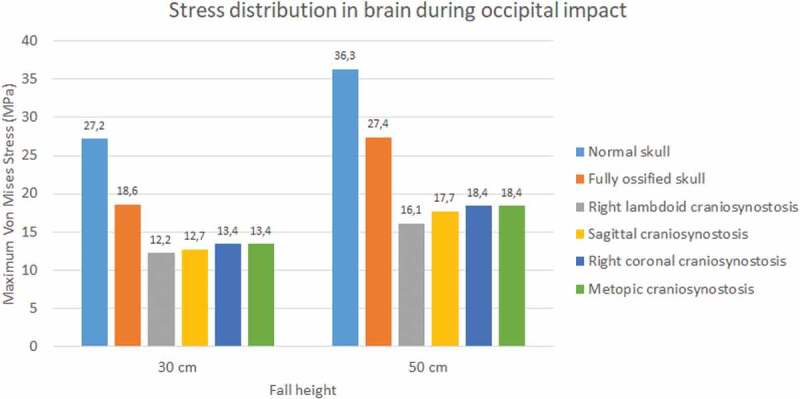
Maximum Von Mises stress in the brain during occipital impact from 30 and 50 cm falls with different degrees of ossification in the sutures

**Figure 18.**
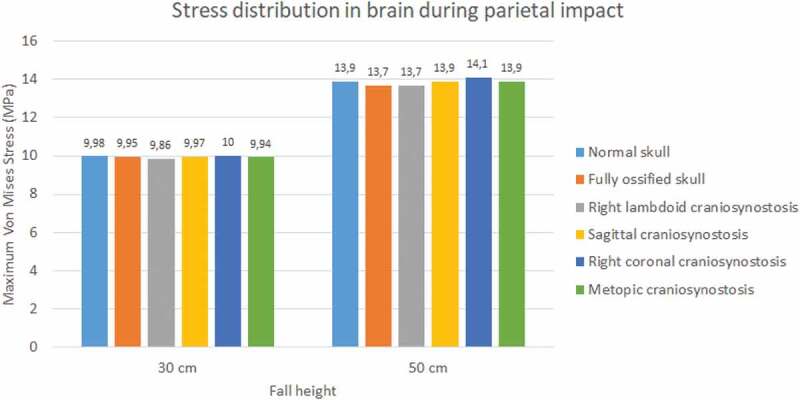
Maximum Von Mises stress in the brain during parietal impact from 30 and 50 cm falls with different degrees of ossification in the sutures

The normal skull showed the highest values of Von Mises stress in occipital impact (27.2 KPa at 30 cm fall and 36.3 KPa at 50 cm fall) followed by the ossified skull (18.6 KPa at 30 cm fall and 27.4 KPa at 50 cm fall) (see Figure 17). The lowest values were found for right lambdoid craniosynostosis (12.2 KPa at 30 cm fall and 16.1 KPa at 50 cm fall). In parietal impact, no significant differences were found between the different cases (see Figure 18). On average, Von Mises stress was equal to 9.95 KPa at 30 cm fall and 13.9 KPa at 50 cm fall.

**Figure 19.**
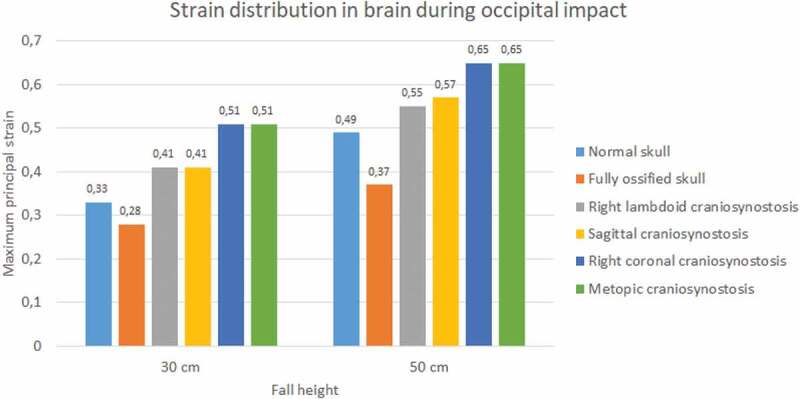
The maximum principal strain in the brain during occipital impact from 30 and 50 cm falls with different degrees of ossification in the sutures

**Figure 20.**
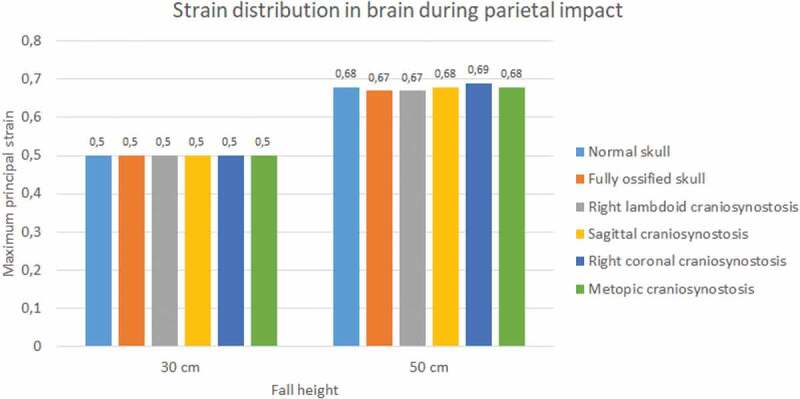
The maximum principal strain in the brain during parietal impact from 30 and 50 cm falls with different degrees of ossification in the sutures

Significant differences were observed in strain during occipital impact (see Figure 19). At both heights, the cases of right coronal and metopic craniosynostosis obtained the highest strain values (0.51 at 30 cm fall and 0.65 at 50 cm fall for both). The lowest values were those obtained by the normal skull (0.33 at 30 cm fall and 0.49 at 50 cm fall) and the ossified skull (0.28 at 30 cm fall and 0.37 at 50 cm fall). In parietal impact, no significant differences were found between the different cases (see Figure 20). On average, the strain in the brain was equal to 0.5 at 30 cm fall and 0.68 at 50 cm fall.

## 4. Discussion

### 4.1. Model validation

Few computational studies concerning head 3D modeling have been experimentally validated. This situation is a result of the scarce experimental evidence on the biomechanical behavior of the head when different mechanical events take place. In this study, quantitative validation of head acceleration results in occipital and parietal impacts due to 30 cm falls was made by comparison with experimental data obtained by (Prange et al. 2004). The results obtained from the finite element model of the neonatal head correlated relatively well with the results of the experimental study made by Prange et al., as shown in Figures 5–6. However, some discrepancies could be noticed. The first one was the impact site. The experimental model defined an immovable impact site, but the actual site could vary between experimental runs. Hence, variations on impact point could lead to fluctuations in the accelerations obtained for each experiment, given by the measured standard deviation. In both studies, the accelerations were calculated by dividing the force of impact by the total mass of the head. This calculation might not have been the most appropriate since acceleration could vary significantly in its maximum value depending on the calculation site (center of mass, impact site, among others). Likewise, model acceleration results showed that the head model was a bit more rigid than the skulls of the experimental study. This result might have been due to different factors. Among them, greater rigidity could be related to the tetrahedral mesh used, which, by its characteristics, tends to show a higher rigidity than a hexahedral mesh. In addition, model skull mass was equal to 629 g, which was not very far from the experimental masses of 650 g used in Prange study on the anthropometry of an infant’s head. Still, by being smaller, it could increase model accelerations by 3.2% when compared to the ones found in the experimental study.

### 4.2. Finite elements model simulation

**Figure 21.**
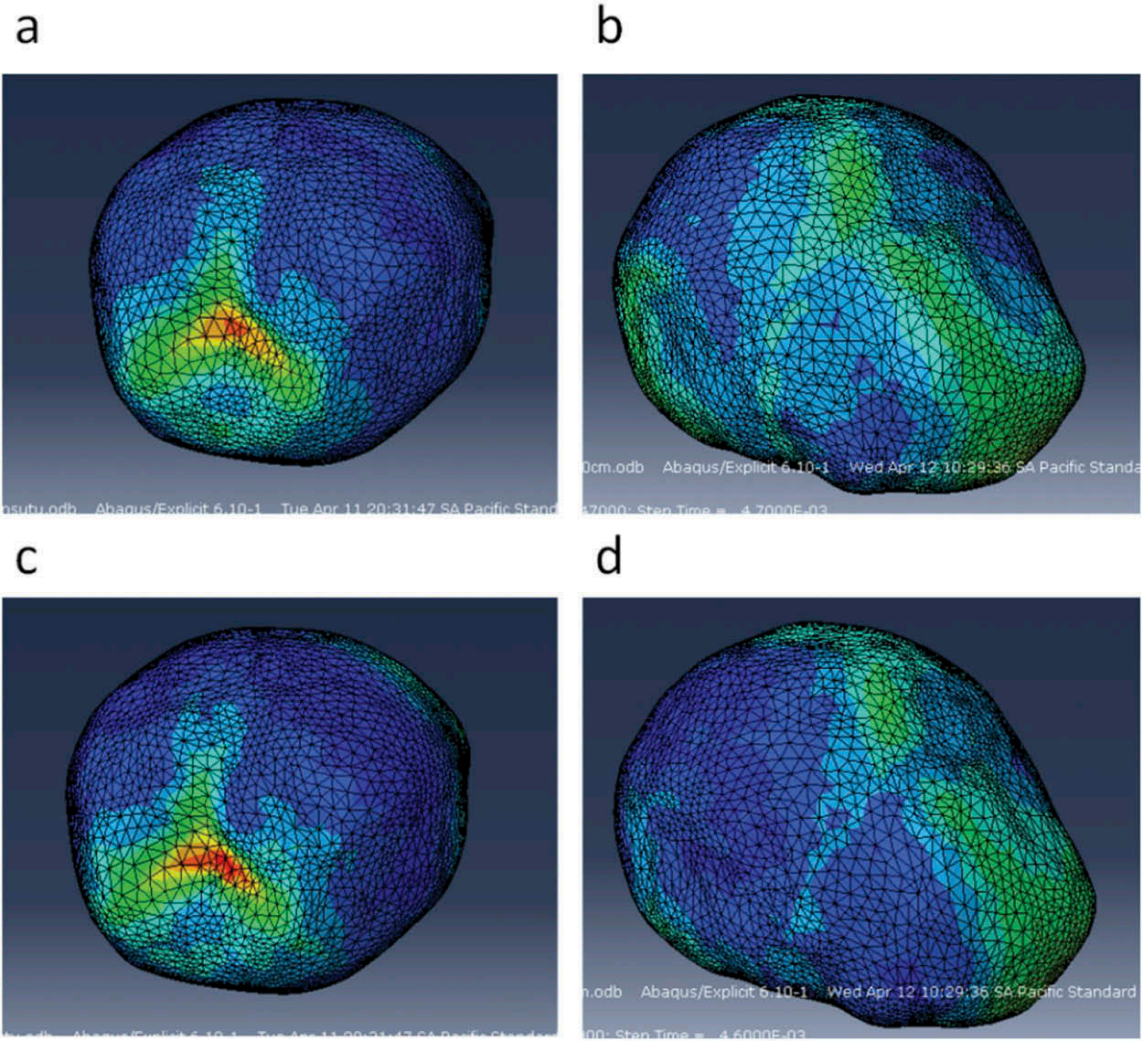
Sites of stress and strain concentration in the brain after occipital and parietal impacts from 30 cm falls. a) Von Mises stress in the brain after occipital impact. b) Von Mises stress in the brain after parietal impact. c) Strain in the brain after occipital impact. d) Strain in the brain after parietal impact

The variation of fall height and the level of suture ossification significantly influenced the mechanical response of bones, brain, and sutures. Impact forces were higher in 50 cm falls than in 30 cm falls. Similarly, stress and strain values in all tissues were higher as fall height increased. All impact simulations showed higher strains with unossified sutures, given by its lower Young’s modulus (8.1MPa) compared to the ossified ones. For simulations with unossified sutures, the occipital impact was localized, and its effect on the brain was in a location similar to the impact site. That is, the maximum Von Mises stress and strains in the brain were found in locations near the impact site on the flat bones. In parietal impact, sites of higher Von Mises stress and strain in the brain were found in the vicinities of the anterior fontanel (the one joining the metopic, coronal, and sagittal sutures). Hence, it could be inferred that lateral impacts are harder, cause higher strains, and the lesion is oriented more towards the anterior fontanel than towards the impact site. Although the impact was on one side of the skull, stress, and strains are higher in the anterior fontanel (see Figure 21).

It was also found that the skull with unossified sutures was very sensitive to low height impacts. An occipital impact caused by falls of up to 30 cm could cause a high concentration of Von Mises stress in the brain (27.2 KPa and 36.3 KPa at 30 and 50 cm falls), which, according to the results, was superior to the one found in skulls with totally ossified sutures and those with some type of craniosynostosis (see Figure 17).

Different degrees of ossification given by the presence of craniosynostosis significantly influenced the stress and strain results in each tissue. The presence of craniosynostosis increased the stress and strain absorption in bones and sutures but did not have a significant effect on stress and strain absorption in the brain. Cases such as right lambdoid craniosynostosis increased stress absorption in bone tissue, which could be related to the reduction of von Mises stress in the brain when compared to impacts on skulls with normal unossified and fully ossified sutures. This result could be due to the increased stiffness acquired by the suture when it becomes fully ossified. Thus, the suture could shield the brain to some extent in the area of occipital impact, which, as noted, is the same area where the greatest Von Mises stress was found in the brain.

Although impact forces were higher in parietal impacts, a more significant stress transfer to the brain was observed in the occipital impact. This result could have been due to the location of the impact, which occurred at the intersection of two sutures (lambdoid suture and sagittal suture), a site with a high degree of strain due to the presence of soft, ductile tissue. However, the former is contrasted with results found for strain. Brain strains during parietal impact were higher than those found in this tissue during the occipital impact. Therefore, when analyzing these damage indicators, it is possible to conclude that both types of impact can be harmful and cause injury, but through different biomechanical mechanisms. For this reason, injury mitigation strategies should be implemented for low height impact protection in neonates. These should be tailored to each specific situation, since impact location might produce different effects on brain tissue, leading to varying degrees of injury depending on the location, height and ossification characteristics of the sutures in the individual.

### 4.3. Limitations and future work

The main limitation of this study was the lack of experimental data concerning the response of the brain, sutures, and bones to the received biomechanical impact. Material properties used for the brain, bones, CSF, and sutures corresponded to those reported by previous numerical studies, which were determined by numerical optimization. Thus, it is necessary to carry out additional biomechanical studies in this research theme to achieve greater precision in the properties, which are determinants of the veracity of numerical results.

The viscoelastic nature of sutures was not considered in this work. In high impact situations, shock waves are rapidly attenuated when viscoelastic behavior is present. Linear elastic models often result in an overall stiffer model, as results from this work could also suggest. This is due to the linear elastic models not allowing higher deformations, which could reduce overall strains in underlying tissues. Nevertheless, even though mature sutures are capable of absorbing more strain energy to failure than cranial bones during an impact, infant skull sutures absorb less energy before failure. Hence, sutures are not capable of absorbing high impact loads in infants, so a viscoelastic model might not improve the accuracy of skull impact dynamics. It should be noted that the highest stress during impact analysis occurs immediately after contact between the rigid surface and skull bones and suture happens. Therefore, viscoelastic models could provide information in instances farther away from this time frame about stress attenuation and diffusion throughout the adjacent tissues in the skull.

The computational study did not contemplate the inclusion of the meninges (dura mater, pia mater, among others) or other anatomical regions of the brain such as the cerebellum, brainstem, gray matter, white matter, among others. This was due to the lack of medical images that detailed the morphology of these tissues with adequate precision to perform the 3d reconstruction of them. Therefore, it is necessary to generate new technological tools that allow the anatomical reconstruction of these tissues in neonates so that they can be effectively incorporated into a new, more complete model of the neonatal skull.

## 5. Conclusions

In this work, we developed a three-dimensional finite elements model of a four-week-old infant head. The model was designed using a STL file that contained the three-dimensional reconstruction of the flat bones that made up the calvaria and served as a template for modeling sutures, brain, and CSF. Validation of the results of the simulations was carried out by comparing the accelerations obtained in the impact simulations with experimental data concerning occipital and parietal impacts due to the free fall of babies of 1, 3, and 11 days old. The model proved to be reliable in following the mechanical behavior reported in the experiments.

Moreover, the developed model was used to evaluate the effect of fall height and sutures ossification level in the biomechanical response of flat bones, sutures, and brain to impact. The results show that, at higher heights, greater impact forces are generated, mainly in parietal impacts, where the fully ossified skull shows the highest impact force. The presence of right lambdoid craniosynostosis presented the highest stress in the flat bones, which could induce a higher probability of fracture in comparison with other types of craniosynostosis. Excluding the fully ossified skull, all cases showed high values of stress and strains in the sutures, which can be an indicator of structural damage in impacts from a low height fall. In all simulated conditions, high values of Von Mises stress were observed in the brain, and high strains were observed in the right and metopic coronal craniosynostosis.

The intrinsic difficulty of experimentation in this area has generated a lack of experimental data regarding the mechanical properties of the different tissues that make up the infant head, as well as a limited number of experiments aimed at obtaining knowledge of the effects of impact dynamics in the etiology of TBI in neonates. For this reason, the improvement of computational models like the one found in this work could allow the development of new strategies aimed towards a greater understanding of the condition and the generation of measures to prevent the damage caused by it.

## Data Availability

The data described in this article are openly available in the Open Science Framework at DOI:10.17605/OSF.IO/TPA6U.
